# High-intensity interval training produces a significant improvement in fitness in less than 31 days before surgery for urological cancer: a randomised control trial

**DOI:** 10.1038/s41391-020-0219-1

**Published:** 2020-03-10

**Authors:** J. E. M. Blackwell, B. Doleman, C. L Boereboom, A. Morton, S. Williams, P. Atherton, K. Smith, J. P. Williams, B. E. Phillips, J. N. Lund

**Affiliations:** 1 University of Nottingham, Nottingham, UK; 2 Royal Derby Hospital, Derby, UK

**Keywords:** Prostate cancer, Cancer therapy

## Abstract

**Objectives:**

To assess the efficacy of high-intensity interval training (HIIT) for improving cardiorespiratory fitness (CRF) in patients awaiting resection for urological malignancy within four weeks.

**Subjects/patients and methods:**

A randomised control trial of consecutive patients aged (>65 years) scheduled for major urological surgery in a large secondary referral centre in a UK hospital. The primary outcome is change in anaerobic threshold (VO_2AT_) following HIIT vs. standard care.

**Results:**

Forty patients were recruited (mean age 72 years, male (39): female (1)) with 34 completing the protocol. Intention to treat analysis showed significant improvements in anaerobic threshold (VO_2AT_; mean difference (MD) 2.26 ml/kg/min (95% CI 1.25–3.26)) following HIIT. Blood pressure (BP) also significantly reduced in following: HIIT (SBP: −8.2 mmHg (95% CI −16.09 to −0.29) and DBP: −6.47 mmHg (95% CI −12.56 to −0.38)). No reportable adverse safety events occurred during HIIT and all participants achieved >85% predicted maximum heart rate during sessions, with protocol adherence of 84%.

**Conclusions:**

HIIT can improve CRF and cardiovascular health, representing clinically meaningful and achievable pre-operative improvements. Larger randomised trials are required to investigate the efficacy of prehabilitation HIIT upon different cancer types, post-operative complications, socio-economic impact and long-term survival.

## Introduction

Urological malignancy is common, with over 69,000 new diagnoses for prostate, kidney and bladder cancer in 2015 in the United Kingdom (UK) alone [1]. Surgery for these cancers is associated with high complication rates (cystectomy 56%, nephrectomy 21%, prostatectomy 19%) [2] and survivors commonly experience fatigue, reduced physical ability and reduced quality of life [3]. To exemplify this, only 30% of survivors have returned to baseline levels of physical function at 3 months after radical prostatectomy (RP) [4] and 50% by 12 months [5]. This reduced physical capacity leads to prolonged time off work [6], predicts early retirement for those in work before operation [7], and contributes to long-term reductions in health-related quality of life (HRQOL) [3, 5] in these patients. As such, recent work has suggested that measures of surgical success should include return to pre-operative levels of quality of life and physical function [8].

Cardiopulmonary exercise testing (CPET), the gold standard clinical measure of cardiorespiratory fitness (CRF) [9], can help to identify those who are least fit and therefore most at risk of post-operative complications, with the complimentary notion being that if CRF can be improved before surgery then outcomes may also improve. In support of this notion, previous studies in general surgery have deemed the minimal clinically important difference (MCID) to be a pre-operative increase in anaerobic threshold (VO_2AT_) of 1.5–2.0 ml/kg/min [10, 11]. Indeed, achieving the MCID was associated with a 40% reduction in the odds of post-surgical complications in colorectal patients [10], which could hold true in urological populations also. In addition, higher levels of pre-operative physical activity are related to better HRQOL scores post-operatively [12], with cancer patients who exercise following diagnosis having a lower relative risk of cancer mortality, cancer recurrence and adverse effects from disease and treatment across all cancers [13].

Prehabilitation exercise regimes aim to increase a patient’s physiological reserve before surgery [14] and have been shown to improve functional fitness post operatively [15]. However, within urology most prehabilitation studies have focussed upon reducing specific urological complications (e.g. urinary incontinence) [16] and not on general physiological parameters known to be associated with improved post-operative outcomes and return to pre-operative status such as CRF [14], skeletal muscle mass [17] and body composition [18]. One consideration for all cancer prehabilitation regimes largely regardless of endpoint is that in the UK, the National Cancer Action Team specifies that first treatment (including surgery) should start within 31 days of the decision to treat [19]. This leaves only a short time-window in which to deliver a prehabilitation intervention and elicit change.

In relation to improving CRF, various forms of exercise training have been used in healthy and clinical populations, with moderate continuous (aerobic) training (MCT) the modality most commonly employed. However, most forms of MCT take too long to elicit a beneficial effect on CRF to be useful in patients waiting for surgery for cancer [20]. High-intensity interval training (HIIT) has been proven effective for improving the CRF of both healthy individuals and clinical populations (including certain cancer groups (e.g. lung, bowel and breast)) over shorter time-periods than needed for MCT [20] and as such has potential utility as cancer prehabilitation. Although there are a number of different HIIT protocols, all combine high-intensity exertions with rest periods [21]. Given that time is often cited as a major barrier to exercise training adherence and compliance [22], HIIT regimes with shorter exercise sessions (i.e. low-volume HIIT) may be the most effective in older patient groups.

The primary objective of this study was to investigate whether patients with urological malignancy could achieve the MCID (VO_2AT_ improvement 1.5–2 ml/kg/min) in CRF in response to HIIT, within a window compliant with UK cancer waiting time targets (31 days). Secondary objectives included the effect of HIIT upon blood pressure, body composition, measures of muscle architecture and patient perceived acceptability of the HIIT protocol used.

## Subjects/patients and methods

This parallel randomised control trial (1:1 allocation ratio) recruited from August 2016 to June 2018 after ethical approval by the NHS research ethics committee (REC reference: 16/EM/0075, IRAS Project ID 19141). This study was written in accordance with CONSORT guidelines [23] and prospectively registered with Clinical trials.gov (NCT02671617).

Patients identified at a weekly multidisciplinary team (MDT) meeting, where a decision to operate for urological cancer had been made, were approached by the research team after being informed of their treatment plan in the outpatient clinic. Patients were only approached if the window between MDT decision to treat and their allocated operation date allowed potential for baseline assessment, 10 or more HIIT sessions and reassessment within 72 h before operation. Adherence to the intervention was defined as completing at least ten HIIT sessions.

Patients first received an information sheet and if interested were invited to a study familiarisation The provided written informed consent and underwent medical screening (performed by medically qualified doctor in line with the eligibility criteria for CPET testing defined by the American Thoracic Society [24]). Randomisation was to either the control group (CON; consisting of standard care) or a four week fully-supervised HIIT intervention (HIIT) using a computer-generated list of random permuted block sizes, stratified according to age and gender (to ensure a higher likelihood of equal baseline characteristics). Allocation concealment was ensured by using opaque, sealed envelopes with participant group allocation performed on the first study visit. Due to the nature of the intervention, patients were not blinded to their group assignment, however data collectors were.

At assessment sessions, participants completed the following questionnaires: the Dukes Activity Status Index (DASI) [25], the EuroQol Group 5-level (EQ-5D-5L) [26] and the Warwick Edinburgh Mental Wellbeing Scale (WEMWBS) [27]. Whole-body composition was measured by dual energy X-ray absorptiometry (DXA), after which muscle architecture (muscle thickness, pennation angle and muscle fascicle length) of the *vastus lateralis was* measured using B-mode ultrasonography [28]. CPET (Lode Corival, Lode, Groningen) was performed as described previously [29], with inline breath-by-breath data collected via a metabolic cart (nSpire Zan 600, Germany), with patients encouraged to exercise to volitional exhaustion [24]. CPET interpretation was conducted by two experienced assessors blinded to time-point (i.e. pre or post-intervention) and group allocation. VO_2PEAK_ (volume of oxygen consumed at the maximal exertion during the test) values were taken as the highest reading in the last 20 s of the test, with VO_2AT_ (volume of oxygen consumed at the anaerobic threshold) determined using a modified V-slope and ventilatory equivalents method [29].

Both HIIT and CON groups were instructed to maintain their habitual physical activity and dietary regimes for the duration of the study. The CON group had assessment sessions 1 and 2 four weeks apart with no visits in between. After assessment session 1, the HIIT group was scheduled for up to 12 HIIT sessions (3–4 times weekly, with no training at weekends) within a 4-week period (i.e. <31 days). The HIIT sessions were delivered on an individual basis at a university exercise laboratory, fully-supervised by a medically qualified doctor. The HIIT protocol was performed on a cycle ergometer and comprised a 2-min warm-up period of unloaded cycling, followed by 5, 1-min exertions at 100–115% of the maximal load (watts (W)) reached during their initial CPET, ending with a 2-min recovery period of unloaded cycling. An increase in wattage was implemented at the mid-way point of training to maintain exercise intensity with progression [29].

Assessment session 2 occurred a maximum of 72 h before surgery for all participants, with participants in the HIIT group also asked to complete a questionnaire about the acceptability of the intervention. This questionnaire has been used previously to assess the acceptability of the same HIIT protocol used in this study [29].

### Statistical analysis

An a priori sample size calculation, using data derived from previous work from our group (ANOVA; partial η2 0.15 (effect size 0.42), for a power of 0.80 and α level of 0.05) indicated that 40 patients in total (20 CON group, 20 HIIT group) would be required to detect a difference in VO_2AT_ of 2 ml/kg/min, with the correlation for repeated measures assumed to be 0.7. Including a 20% attrition rate (based on drop-out rates in similar studies) the maximum recruitment number was 48 patients. Descriptive data is presented as mean (±SD), median [inter-quartile range (IQR)] and number (%) as appropriate. To analyse outcomes, we used ANCOVA with pre-intervention baseline values as continuous covariates. Normality was assessed using histograms, and scatter plots were used to assess the relationship between outcomes and covariates. We assessed equality of variance using variance comparison tests. For correlations, we used Pearson’s and Spearman’s as appropriate. Modified intention to treat was conducted on all participants randomised and who underwent both assessment sessions and sensitivity analysis was also performed on prostate cancer patients completing a minimum of ten HIIT sessions. All analyses were conducted using Stata Version 15.1.

Public involvement within the design of this study was during the ethical review process only.

## Results

**Fig. 1:**
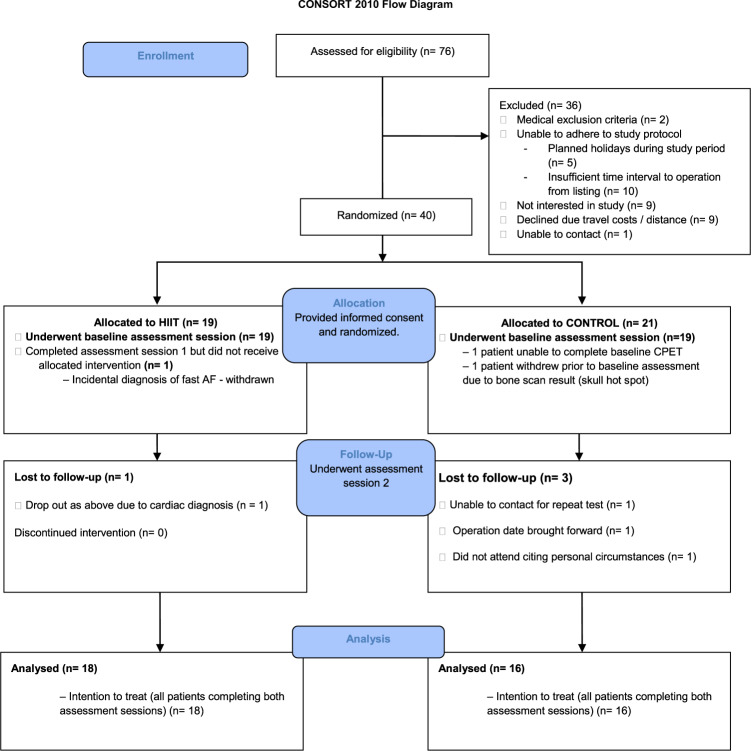
Consort diagram showing patient flow through study [19]. *HIIT* high intensity interval training, *CPET* cardiopulmonary exercise testing, *AF* atrial fibrilation.

Forty patients were randomised during the study period (Fig. 1). Statistical analysis for the primary outcome measure (VO_2AT_) was conducted at the end of the ethically approved study period. Baseline characteristics for both groups can be seen in Tables 1 and 2. For those patients randomised to HIIT, adherence (ten or more HIIT sessions) to the exercise training protocol was 84% (16/19). Of those who did not adhere one dropped out of the study, one participant did not attend a single scheduled HIIT session, and one participant only completed six sessions due to a planned holiday. Patients initially trained at 100–115% of maximum wattage achieved at baseline CPET (145 [30] Watts) and all but two patients had a 10% increase in wattage after six sessions as per protocol. The median [IQR] time between baseline and reassessment session for CON and HIIT was 28 [22–29, 31–33] and 30 [27–29, 31] days, respectively. The median [IQR] number of HIIT sessions was 11 [10–12] (excluding those not adherent to the protocol).

**Table 1:** Patient baseline characteristics of individuals before a <31-day control period (CON) or period of high-intensity interval training (HIIT).

Baseline characteristics	CON (*n* = 21)	HIIT (*n* = 19)
Age (years)	72 (4)	71 (2)
Gender (m:f)	20:1	19:0
DASI	47.97 (9.3)	51.38 (9.6)
Weight (kg)	79.6 (14.4)	80.1 (10.4)
CPET Wattage (W)	145 (37)	137 (33)
VO_2PEAK_ (ml/kg/min)	26.4 (5.7)	24.8 (5.2)
VO_2AT_ (ml/kg/min)	13.84 (2.8)	13.15 (1.9)
SBP (mmHg)	144 (12)	139 (13)
DBP (mmHg)	82 (8)	82 (9)

*DASI* Dukes Activity Status Index, *CPET* cardiopulmonary exercise test, *SBP* systolic blood pressure, *DBP* diastolic blood pressure, mean (±standard deviation).

**Table 2:** Patient clinical characteristics of individuals before a <31-day control period (CON) or period of high-intensity interval training (HIIT).

Clinical characteristics	Control (*n* = 21)	HIIT (*n* = 19)
Location of malignancy		
Prostate	17	18
Bladder	2	1
Kidney	2	0
Operation		
RARP	14	14
Open prostatectomy	0	1
Radical cystectomy	2	1
Laparoscopic nephrectomy	2	0
Change to clinical plan	3	3
Co-morbidities		
Hypertension (medicated)	7	8
Diabetes	2	0
Musculoskeletal (osteoarthritis, rheumatoid arthritis or joint replacement)	8	8
None	4	2
Other	Ulcerative colitis, asbestosis, gout, chronic kidney disease, asthma	Chronic fatigue, hypothyroidism (×2), chronic lymphocytic leukaemia, myocardial infarction, coronary artery bypass-grafting, asthma
Pre-op PSA (ng/ml)	9.38 [8.1–13.2]	9.17 [6.9–10.9]
D’Amico Risk Classification for Prostate Cancer [45]		
High risk	7	6
Intermediate	10	10
Low	0	2

Radical cystectomy includes both cystoprostatectomy and anterior exenteration and change to clinical plan after recruitment to study included no operation, watchful waiting and/ or radiotherapy.

*RARP* Robotically assisted radical prostatectomy.

### Cardiorespiratory fitness

**Fig. 2:**
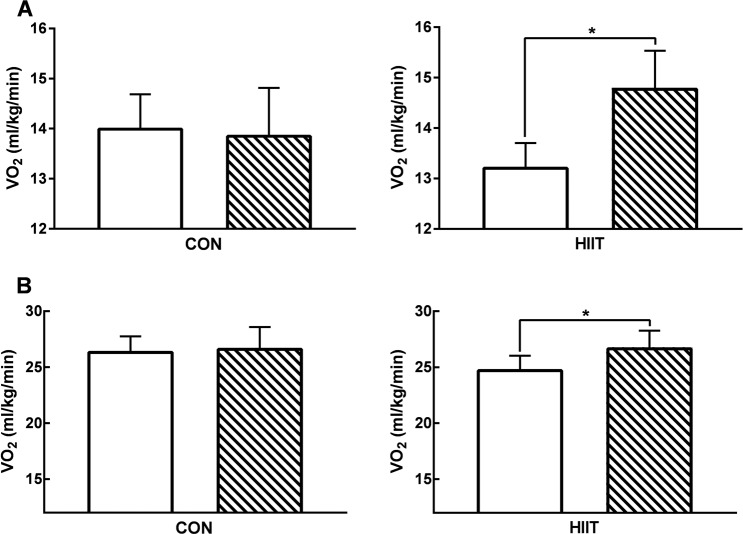
Cardiorespiratory fitness. Baseline (clear) and post intervention (hatched) cardiorespiratory fitness data for individuals before and after a < 31-day control period (CON; *n*  =  16) or period of high-intensity interval training (HIIT; *n*  =  18). **a** Volume of oxygen utilised at anaerobic threshold (VO2AT). **b** Peak volume of oxygen utilisation (VO2PEAK); both measured via cardiopulmonary exercise testing (CPET). *p < 0.05 vs. baseline. Analysis via ANCOVA.

Based on all study participants who completed both pre and post intervention CPET (modified intention to treat analysis), there was a significant improvement in VO_2AT_ (mean difference (MD) 2.26 ml/kg/min (95% CI 1.25 to 3.26)) and VO_2PEAK_ (MD 2.16 ml/kg/min (95% CI 0.24 to 4.08)) following HIIT (Fig. 2). Similarly, there was a significant increase in CPET wattage at failure in the HIIT group (MD 12.86 W (95% CI 5.52–20.19)). In the HIIT group neither VO_2PEAK_ (*r*^2^ = 0.02, *p* = 0.60) nor VO_2AT_ (*r*^2^ = 0.04, *p* = 0.43) improvements were correlated with baseline values. Sensitivity analysis of prostate cancer patients alone (all 15 patients with prostate cancer adhered to the HIIT protocol) showed a significant improvement in VO_2AT_ of 2.19 ml/kg/min (95% CI 1.06–3.32) following HIIT.

### Cardiovascular health

**Fig. 3:**
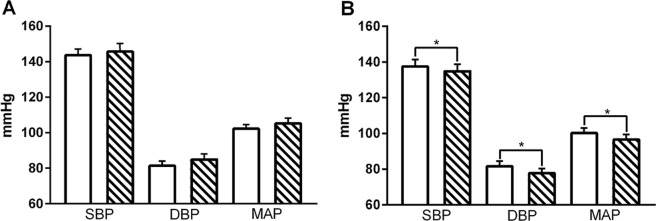
Blood pressure. Baseline (clear) and post intervention (hatched) non-invasive blood pressure readings (SBP, DBP, MAP; mmHg) for individuals before and after a < 31-day control period (CON; **a** *n* = 16) or period of high-intensity interval training (HIIT; **b**, *n* = 18). *p < 0.05 vs. baseline. Analysis via ANCOVA.

There was a statistically significant reduction in resting blood pressure (BP) parameters following: HIIT (systolic blood pressure (SBP): −8.2 mmHg (95% CI −16.09 to −0.29), diastolic blood pressure (DBP): −6.47 mmHg (95% CI −12.56 to −0.38)) with no change in either parameter in the CON group (Fig. 3)).

### Body composition and muscle architecture

Ultrasound image analysis of the *m. vastus lateralis*, showed significant increases in muscle thickness (MT; MD 0.22 mm (95% CI 0.02 to 0.41)) and pennation angle (PA; MD 2.49 degrees (95% CI 0.42–4.55)) in the HIIT group. Muscle fascicle length (FL) was not altered in either group (HIIT, MD −0.08 mm (95% CI −0.87 to 0.71)). There were no significant changes in any DXA derived parameter of body composition following HIIT (total weight: −0.25 kg (95% CI −1.07 to 0.57)), total body fat percentage: 0.13 % (95% CI −0.64 to 0.90) or total lean mass: −230.9 g (95% CI −1156.10 to 694.16).

### Safety and acceptability

There were no adverse safety events reported throughout the study. Mild leg pain at the end of exercise and discomfort from the cycle ergometer seat were reported by two individuals, both of which were self-limiting and required no intervention. All participants were able to achieve >85% maximum predicted heart rate during the HIIT sessions.

There were no significant changes in WEMWBS (−0.88 (95% CI −4.17 to 2.4)), EQ-5D-5L overall health score (visual analogue scale) 3.65 (95% CI −1.97 to 9.28), or 5D-EQ-5L index values (−0.03 (95% CI −0.08 to 0.02)) following HIIT.

Patients in this study reported our HIIT protocol to be enjoyable and highly acceptable, they would recommend HIIT to others, and despite there being no significant changes in the quality of life-based questionnaires, patients were pleased to have improved their own fitness (Table 3). Over half of the participants attended sessions with a spouse or family member who did not participate in the intervention. Specific data sets relating to this results section may be requested from the corresponding author.

**Table 3:** HIIT acceptability questionnaire results.

Statement	Median score [IQR] 5: Strongly agree 1: Strongly disagree
HIIT was well explained	5 [Scored by all]
I enjoyed HIIT	5 [Scored by all]
HIIT was a time burden	1.5 [1,2]
I would recommend HIIT to others	5 [Scored by all]
HIIT was more demanding than expected	3 [2–4.5]
I would do HIIT again	5 [4,5]
The travel associated with HIIT interfered with my life	1.5 [1–2.5]
The physical strain interfered with my life	2 [1–3]
I believe my fitness has improved	5 [Scored by all]
I am pleased to have done something to improve my fitness	5 [Scored by all]
I would like to have exercised in a group	1 [1,2]
I would like to have exercised at home	1 [1–2.25]

## Discussion

Patients with urological cancer can make clinically meaningful improvements in objectively measured CRF (VO_2AT_ and VO_2PEAK_) within 31 days, prior to surgery. As previously shown in healthy volunteers [29] and a different cancer cohort [31], our 5 by 1-min HIIT protocol can be safely delivered, and was reported to be enjoyable by our specific patient group. A number of physiological principles support the implementation of either unimodal or multimodal pre-operative interventions in patients diagnosed with cancer requiring surgical intervention [14] and this study provides evidence for a feasible and efficient unimodal exercise protocol that is effective within urological (predominantly prostate cancer) patients. That we successfully improved CRF within the UK cancer target waiting times of 31 days from decision to treat is important for both feasibility and generalisability, as 93% of urological cancer patients receive first definitive treatment within 31 days of decision to treat (data for the second quarter of 2018) [32]. Individualised supervised laboratory-based HIIT is resource (equipment and staffing) dependent intervention which would likely limit its pragmatic delivery in this format, but does allows monitoring of adherence to the prescribed HIIT sessions and confirmation that high-intensity (>85% heart rate maximum) exercise was achieved during every individual session. Based on previous work, the exercise intensity likely contributed to the significant gains in CRF seen within this study [33], but this intensity and compliance is hard to deliver in unsupervised exercise regimes [30]. We also report significant reductions in BP following just 1-month of HIIT, suggesting HIIT to be more potent than traditional aerobic exercise training where reductions of 5 mmHg in SBP and 3 mmHg in DBP over a 3-month period are reported [34].

Although less objective measures of functional fitness (e.g. improved 6-min walk test (6-MWT) and increased muscle leg power) have been shown to improve within a 31-day timeframe in patients with cancer [35, 36], it has previously proved difficult to improve VO_AT,_ (as measured by CPET) by the MCID (1.5–2.0 ml/kg/min) for all comers in major benign [37] and malignant intra-abdominal surgery [11, 31, 38] using supervised exercise regimes. In addition, until recently, prehabilitation studies in urological patients have largely been aimed at reducing specific urological complications (e.g. urinary incontinence) and this has been successfully achieved with pelvic floor muscle training [16]. However, given the well-reported post-operative decline in physical functioning and reduced HRQOL scores following prostatectomy [3, 5], this study’s findings of improved CRF, reduced BP and positive adaptations in skeletal muscle architecture suggest that our HIIT regime, when used as prehabilitation, has the potential to reduce peri-operative morbidity and mortality, and via pre-operative improvements in strength and fitness, may improve time to return to baseline function after surgery [14, 15].

Although multimodal prehabilitation has shown promise, the efficacy of individual components of these regimes can be difficult to quantify. In addition, exercise prescription as a unimodal intervention has produced mixed outcomes for patients with cancers at different primary sites [11, 20], resulting in a lack of consensus on the optimal training modality and regime. In one urology prehabilitation study, patients awaiting RP who participated in home-based mixed-modality (aerobic exercise, resistance exercise training and pelvic floor training) exercise training did show improvements in 6-MWT before surgery and a more rapid return to pre-operative walk test distances after surgery compared to control [39]. Although this study used different outcome measures to those assessed by us (6-MWT vs. VO_2_ parameters), it would seem that the presence of prostate cancer does not inhibit positive physiological adaptations to exercise training, adaptations which may be blocked by the presence of other types of cancer [40]. Although it is not entirely clear why patients with certain cancers would be less responsive to HIIT, impaired metabolic processes leading to anabolic blunting (reduced mitochondrial enzyme activity and muscle protein synthetic responses to anabolic stimuli) as seen with colorectal cancer in situ [41] may be less pronounced in prostate cancer. It is hypothesised that HIIT may even mitigate this pro-inflammatory environment induced by cancer [42].

In addition to improvements in CRF, we have also shown favourable changes in muscle architecture of the *m. vastus lateralis* that are similar in magnitude to those previously seen in healthy older individuals in response to both resistance exercise training [43] and HIIT [29]. Increasing the quality of skeletal muscle (thickness and pennation angle) increases the potential maximal force generating capacity of the muscle [43], which may aid rehabilitation and promote a quicker return to baseline physical function after surgery.

Our HIIT protocol was highly acceptable and enjoyable for the urological cancer patients within this study, although a number of factors outside the exercise regime may have influenced this finding. For example, a number of our patients brought family members or spouses to the HIIT sessions, with previous research reporting quality of life scores in prostate cancer survivors to be significantly associated with the degree of outcome satisfaction among both the patients and their spouses or partners [3]. In addition, our HIIT protocol was delivered in an exercise laboratory environment supervised by a doctor. These factors related to facilities and supervision may also have influenced the self-reported enjoyment of HIIT in this study, and it is as yet unclear how changing elements of our protocol delivery (i.e. unsupervised HIIT) would affect the reported enjoyment and acceptability levels.

### Study limitations

The primary limitation of this study relates to the homogeneity of the sample; the majority had prostatic adenocarcinoma, and were white males. Although this specific group of men are clearly trainable, making significant gains in a range of cardiorespiratory parameters comparable to healthy counterparts of a similar age [29], this homogeneity will impact the broader applicability of these findings. Exercise studies are, by their inherent nature, at risk of selection bias as all participants have to be willing to be randomised to the HIIT arm of the study. Although participants were instructed to maintain their habitual activity levels and normal dietary intake throughout the study, we did not measure this in either group, with heightened habitual physical activity a possible contributor to the improvements in CRF seen in the HIIT group. Similarly, alterations in dietary intake may have impacted aspects of this study, especially those related to skeletal muscle mass, given the known importance of contraction × nutrition interactions for optimal anabolism. Referrals to our centre come from a wide geographical area, and eligible patients cited travel time and potential financial cost incurred by travelling from home to the exercise laboratory as barriers to participation. Finally, although post-operative complications (as per the Clavien Dindo classification system (CD) [44]) are commented upon for completeness, the study was not powered for formal analysis of this and as such should be interpreted with caution. Within the HIIT group one patient undergoing radical cystectomy suffered a 4b CD complication due to urosepsis and went on to have an intensive care admission. One patient was readmitted with vomiting and was found to have a port site hernia which was surgically repaired (CD 3b). One patient was transfused red blood cells due to a port site bleed which did not require further intervention (CD 2). Two further patients suffered CD 1 complications including additional analgesia requirement and oral steroids for endotracheal tube irritation. CD complications recorded for the control group were: two patients suffered grade 2 CD complications (IV antibiotic administration due to urosepsis) with a third patient requiring additional analgesia (CD 1).

### Concluding statement

Pre-operative HIIT improves CRF, cardiovascular health and measures of skeletal muscle architecture, all of which represent a clinically meaningful and achievable improvement in the health status of urological (primarily prostate) cancer patients in the time available before surgery. Further work is required to investigate the generalisability of this finding to a more heterogenous population, including those with different cancer types. Large randomised trials are required to investigate the effect of prehabilitation upon post-operative complications, socio-economic impact and long-term survival following surgery for urological malignancy.
